# HEALS-A and GRADES: Novel Histological and Clinical Scales for Assessing Skin Regeneration in Murine Wound Healing Models

**DOI:** 10.3390/diagnostics15030387

**Published:** 2025-02-06

**Authors:** Jose R. Muñoz-Torres, Idalia Garza-Veloz, Perla Velasco-Elizondo, Margarita L. Martinez-Fierro

**Affiliations:** Molecular Medicine Laboratory, Academic Unit of Human Medicine and Health Sciences, Universidad Autonoma de Zacatecas, Zacatecas 98160, Mexico; jose.rmt26@gmail.com (J.R.M.-T.); pvelasco@uaz.edu.mx (P.V.-E.)

**Keywords:** regenerative, skin, scale, histological, preclinical, murine

## Abstract

**Background:** Wounds affect approximately 15 out of every 1000 individuals, representing a significant healthcare challenge. The preclinical evaluation of novel wound treatments is important for advancing therapies that promote effective skin regeneration and improve healing outcomes. **Methods:** In this study, we integrated existing knowledge from the literature on murine wound healing models, histological features of the skin, and clinical scores described in humans to propose two complementary assessment tools: the HEALS-A histological score (healing, epithelialization, angiogenesis, leukocytes, scar tissue, appendages) and the GRADES clinical score (granulation tissue, redness/edema, appearance of wound, devitalized tissue). **Results:** These scales combine real-time clinical observation with detailed histological analysis, providing a practical and comprehensive approach to assessing wound healing. Unlike existing wound assessing approaches, HEALS-A does not require specialized software and considers regenerated tissue structures, ensuring a broader and more-detailed evaluation. **Conclusions:** The assessment of wound closure over time, combined with clinical evaluation and histological analysis of skin, provides a comprehensive approach to determining the true impact of new treatments on skin regeneration and the recovery of its functions in wounds.

## 1. Introduction

Wounds are defined as any damage or trauma that alters the functional integrity or anatomy of the skin, mucous membranes, or other organs [1,2]. They are a highly prevalent condition worldwide, with 15.03 cases per 1000 people; of these, 10.55 correspond to acute wounds and 4.48 to chronic wounds [3].

The goal of new treatments and devices to treat wounds is to improve the healing process, reduce complications, and prevent chronic wounds [4]. Preclinical assessment (i.e., animal models) is essential for all newly developed treatments for wound healing. In this phase, the objective is to determine their safety, efficacy, and biocompatibility before they are considered for use as a treatments in humans [5].

Both in humans and preclinical models, the wound healing evaluations are carried out macroscopically and microscopically. The first approach involves evaluating the direct effects on wound closure time and healing patterns or clinical response [6,7]. In human studies, clinical scales are often employed to quantify the healing progress and patient outcomes, bridging preclinical findings with observable, standardized metrics in clinical settings. Clinical assessment enables tailored therapeutic planning based on the clinical response of the patient. Tools like “DOMINATE” guide clinicians through key aspects of the wound healing process, aiming to standardize wound management in clinical practice.

The checklist of the DOMINATE acronym includes the following: D (Debridement), O (Discharge), M (Moisture, Malignancy, Medication, Mental Health), I (Inflammation, Infection), N (Nutrition), A (Arterial Insufficiency), T (Advanced Therapies), E (Edema, Education) [7,8]. More recently, the TIMERS framework (Tissue, Infection/Inflammation, Moisture, Wound edge, Repair/Regeneration, Social) was introduced to provide a comprehensive approach to wound evaluation [6]. Additionally, the wound triangle assessment, which evaluates the wound bed, the wound edge, and the perilesional skin, complements these frameworks. Although it was originally developed for diabetic foot assessment, the wound triangle is now applicable across various types of wounds [9,10]. The second approach, which is microscopic, is generally used in animal models and involves histological evaluation of dermal tissue from the wound site that has undergone treatment, assessing scar characteristics, skin regeneration, and underlying problems affecting wound closure [11,12,13]. Most of the time, histological assessments performed on animal wound models rely on software tools to perform measurements of specific histological structures of particular interest to each researcher [14]. Other histological scales focus on the characteristics of the scar formed after different treatments [12,15]. Some works include histological evaluation, along with methods that assess gene expression through RNA or proteins. These molecules include cytokines, growth factors, and genes involved in healing, which will be considered in the following sections [12,16,17].

### 1.1. Classification of Injuries and Physiology of Wound Healing in Preclinical Models

**Figure 1:**
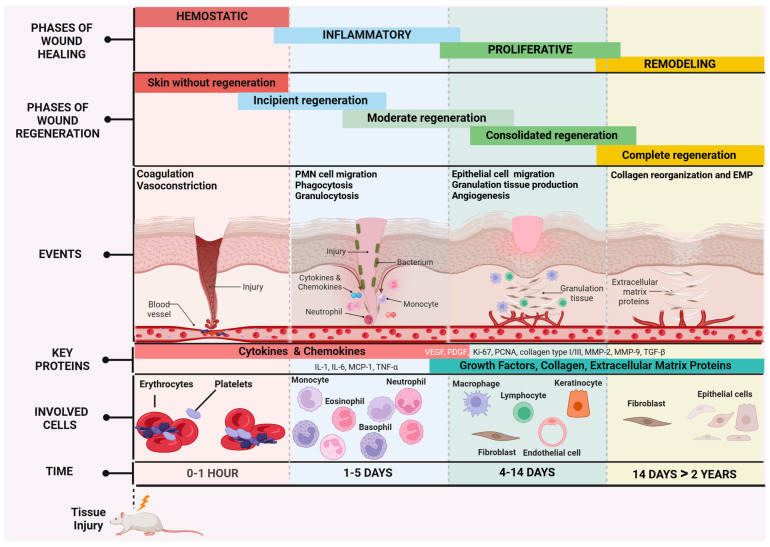
Stages of the wound healing process. The normal wound healing process consists of a series of stages that occur dynamically and sequentially: the hemostatic stage involves platelet aggregation and vasoconstriction; the inflammatory stage, where neutrophils eliminate pathogens by phagocytosis, releases proinflammatory cytokines and chemokines to recruit other leukocytes; in the proliferative stage, where inflammation decreases due to macrophages, angiogenesis occurs, keratinocytes and fibroblasts migrate, and granulation tissue is produced; finally, in the remodeling stage granulation tissue is converted into mature scar tissue through the production of the extracellular proteins (EMPs) and collagen. IL-1: Interleukin-1, IL-6: Interleukin-6, MCP-1: Monocyte Chemoattractant Protein-1, TNF-α: Tumor Necrosis Factor-alpha, VEGF: Vascular Endothelial Growth Factor, PDGF: Platelet-Derived Growth Factor, Ki-67: Proliferation Marker Protein Ki-67, PCNA: Proliferating Cell Nuclear Antigen, collagen type I/III: collagen type I and III, MMP-2: Matrix Metalloproteinase-2, MMP-9: Matrix Metalloproteinase-9, TGF-β: Transforming Growth Factor-beta.

Conventional murine models include incisional and excisional wounds [18]. The incisional model involves wounds that are sutured from the edges. This method allows the development of new suture materials or devices that join the edges of the wound; in this model, it is necessary to evaluate the tensile strength of the skin and the impact on the wound healing process by primary intention [19]. The excisional model is a model where the epidermis, dermis, and subcutaneous cellular tissue are removed without suturing the edges of the wound. This model is used to evaluate therapies that accelerate or improve closure by secondary intention. Considering the former, it is important to evaluate at the histological level the therapeutic effect at the different stages of the skin repair process [20]. In both incisional and excisional models, histological evaluation allows the identification of cellular changes specific to each stage of the wound healing process: (1) hemostatic, (2) inflammatory, (3) proliferative, and (4) remodeling (see Figure 1) [21]. In the hemostatic stage, the blood vessel caliber is reduced, and a clot forms to limit blood loss. Growth factors essential for fibroblast function and dermal stem cell activity are also released [22]. In the inflammatory phase, neutrophils are recruited to eliminate pathogens by phagocytosis [23], neutrophil extracellular traps (NETs), and reactive oxygen species (ROS) [24]. Cytokines and chemokines are released, which promote the recruitment of other leukocytes, making them essential cells for healing [25,26,27]. The proliferative phase, occurring between days three and seven, is characterized by angiogenesis, epithelial migration, and growth at the wound edges, as well as the production of collagen and granulation tissue by fibroblasts [28]. During this phase, macrophages play a key role in resolving the acute inflammatory process while promoting the proliferation of fibroblasts and keratinocytes [29,30]. The remodeling phase corresponds to the maturation of the scar, beginning in the third week and extending up to twelve months [31]. During this phase, the number of capillaries decreases as they integrate into larger blood vessels, and the collagen along with extracellular matrix proteins is reorganized, ultimately restoring up to 80% of the skin’s tensile strength [32].

### 1.2. Molecular Markers Involved in the Wound Healing and Regeneration Process

The presence or absence of molecular markers in wounds depends on factors such as the timing, type of wound, underlying diseases that contribute to or hinder wound healing, clinical response, and other related variables [33,34,35]. The different phases of wound healing overlap, and similarly the expression of various molecules may persist across phases, albeit with some changes in their expression levels (Figure 1) [36,37]. Currently, no single molecule has been identified that can definitively indicate the beginning or end of each phase of wound healing. However, several molecular markers with different functions have been identified, which can assist in a more-thorough evaluation of wounds [38]. These molecular markers can be categorized into early and late-stage indicators, reflecting the dynamic progression of wound healing and/or their functional usefulness. Early stage markers include inflammatory cytokines such as Interleukin-1 (IL-1), Interleukin-6 (IL-6), and Tumor Necrosis Factor-alpha (TNF-α), which initiate the immune response, and growth factors like VEGF (Vascular Endothelial Growth Factor) and PDGF (Platelet-Derived Growth Factor), which promote angiogenesis and fibroblast recruitment. Proliferation markers such as Ki-67 (Proliferation Marker Protein Ki-67) and PCNA (Proliferating Cell Nuclear Antigen) are also critical indicators during the proliferative phase. On the other hand, late-stage markers such as collagen type I/III, MMP-2 (Matrix Metalloproteinase-2), MMP-9 (Matrix Metalloproteinase-2), and TGF-β (Transforming Growth Factor-beta) provide insights into tissue remodeling and maturation. The transition of macrophages from a pro-inflammatory to a reparative phenotype further reflects the resolution of inflammation and tissue regeneration [33,39,40].

**Figure 2:**
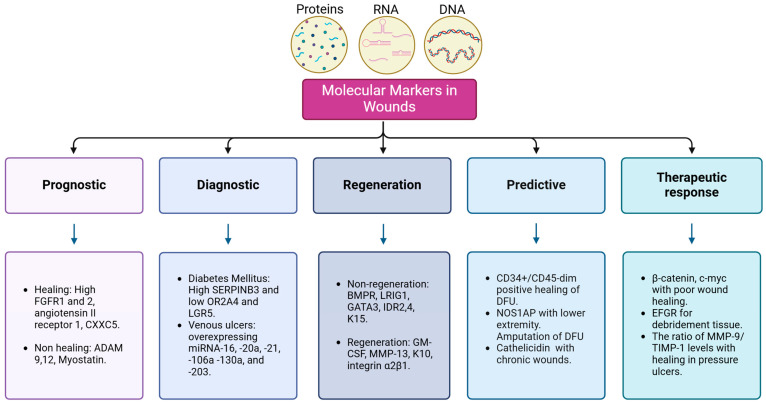
Molecular markers with functional usefulness in wound assessment. There are different types of molecular markers for wound assessment; the researcher must be clear about the purpose of the molecule in order to integrate the result of the molecular marker with complementary evaluations. FGFR1 and 2: Fibroblast Growth Factor Receptor 1 and 2, CXXC5: CXXC-Type Zinc Finger Protein 5, ADAM9 and 12: A Disintegrin and Metalloprotease 9 and 12, SERPINB3: Serpin Peptidase Inhibitor, Clade B, Member 3, OR2A4: Olfactory Receptor Family 2 Subfamily A Member 4, LGR5: Leucine-Rich Repeat-Containing G-Protein Coupled Receptor 5, microRNAs: miRNAs, BMPR: Bone Morphogenetic Protein Receptor, LRIG1: Leucine-Rich Repeats and Immunoglobulin-Like Domains Protein 1, GATA3: GATA Binding Protein 3, ID2 and ID4: Inhibitors of DNA-Binding Proteins 2 and 4, KRT10 and 15: Keratin 10 and 15, GM-CSF2: Granulocyte-Macrophage Colony-Stimulating Factor 2, MMP-13: Matrix Metalloproteinase-13, ITGA2B1: Integrin α2β1, CD34/CD45: Cluster of differentiation 34/45, NOS1AP: nitric oxide synthase 1 adaptor protein, DFU: diabetic foot ulcer, c-myc: cellular myelocytomatosis homologous viral oncogene protein, EGFR: Epidermal growth factor, MMP-13: Matrix Metalloproteinase-13, TIMP-1: TIMP Metallopeptidase Inhibitor 1.

Figure 2 organizes the markers based on their clinical utility and specific role within the process of wound healing assessment and management. In this sense, the diagnosis of some diseases associated with impaired wound healing can be made through high levels of SERPINB3 (Serpin Peptidase Inhibitor, Clade B, Member 3) and low levels of OR2A4 (Olfactory Receptor Family 2 Subfamily A Member 4) and LGR5 (Leucine-Rich Repeat-Containing G-Protein Coupled Receptor 5) molecular markers, which differentiate the skin of patients with diabetes [41]. Venous ulcers are characterized by the overexpression of microRNAs (miRNAs), specifically miR-16, miR-20a, miR-21, miR-106a, miR-130a, and miR-203 [42]. The promotion of healing can be predicted by markers such as the high expression of FGFR1 and FGFR2 (Fibroblast Growth Factor Receptor 1 and 2), AGTR1 (Angiotensin II Receptor Type 1), and CXXC5 (CXXC-Type Zinc Finger Protein 5) [43,44,45], while markers such as ADAM9, ADAM12 (A Disintegrin and Metalloprotease 9 and 12), and MSTN (Myostatin) obstruct the healing process [46,47,48].

Wound regeneration can also be assessed through certain molecular markers, where the low expression of BMPR (Bone Morphogenetic Protein Receptor), LRIG1 (Leucine-Rich Repeats and Immunoglobulin-Like Domains Protein 1), GATA3 (GATA Binding Protein 3), ID2 and ID4 (Inhibitors of DNA-Binding Proteins 2 and 4), and KRT15 (Keratin 15) are markers of poor regeneration [49]. In contrast, CSF2 (Granulocyte-Macrophage Colony-Stimulating Factor), MMP-13 (Matrix Metalloproteinase-13), KRT10 (Keratin 10), ITGA2B1 (Integrin α2β1), and TP63 (Tumor Protein p63) are markers of a good regenerative process [50,51,52]. Other markers have predictive potential, such as CD34+/CD45-dim, which positively predicts the healing of diabetic foot ulcers [53]. Meanwhile, genetic variants of the nitric oxide synthase 1 adaptor protein (NOS1AP; OMIM:60551) have been associated with lower extremity amputation (LEA) [54]. The presence of CTNNB1 (Beta-catenin) and MYC (c-myc) in the epidermis may serve as molecular markers of poor wound healing [55], and decreased levels of CAMP (Cathelicidin) predict wound chronicity [56]. Additionally, the MMP-9/TIMP-1 ratio is a predictor of healing in pressure ulcers [57].

Unlike the standardized and validated clinical scales used for assessing wound healing in humans, animal models lack such consensus tools. Currently, there are no established clinical scales for animals, and wound healing assessments rely largely on individual wound characteristics. In this sense, the wound healing evaluation in animal models requires the adaptation of methodologies to capture the regenerative process effectively due to their faster healing rates and the limitations of existing techniques. Considering the above, this work proposes two tools for preclinical models to evaluate the healing process of excisional wounds or other types of wounds that heal by secondary intention, in which all layers of the skin have been removed. The first tool is a novel histological scale, the HEALS-A scale (healing, epithelialization, angiogenesis, leukocytes, scar tissue, appendages), that allows staging of the degree of skin regeneration during the healing process and identifies wound complications without the need of software tools. This scale reflects the regenerated skin structures and therefore the recovered functionality of the injured skin. Second, we introduce the clinical GRADES scale (granulation tissue, redness/edema, appearance of wound, devitalized tissue), which was developed by adapting a clinical scale into a new tool that allows a comprehensive assessment of new treatments in an animal model of wounds.

## 2. Experimental Design

### 2.1. Histological Scale

#### Histological Evaluation Scale for Preclinical Rodent Models (HEALS-A)

To enhance the evaluation of wound healing in preclinical models, we propose a histological scale designed to assess key aspects of tissue regeneration. We named it the HEALS-A scale (healing, epithelialization, angiogenesis, leukocytes, scar tissue, appendages).

**Figure 3:**
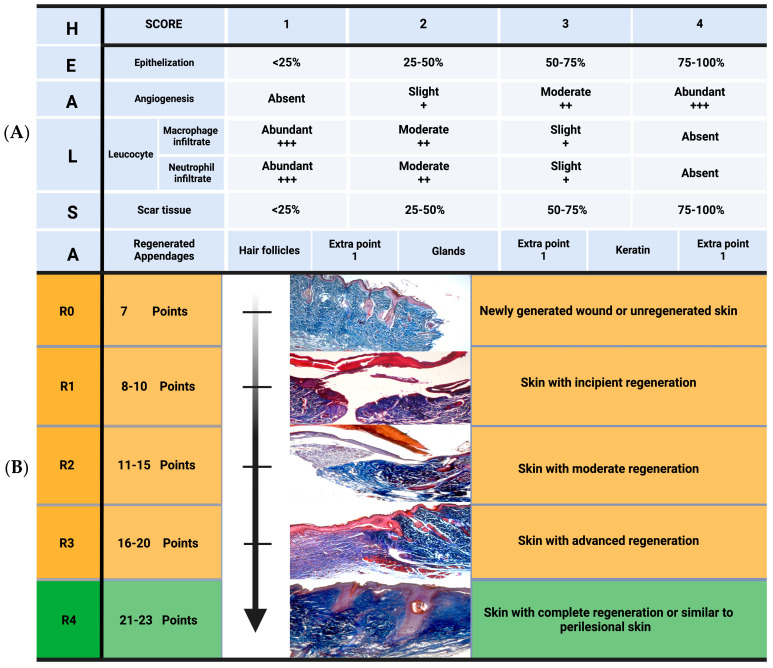
Histological scale of skin regeneration (HEALS-A). (**A**) The histologic features to be evaluated are as follows: epithelialization, scar tissue, new blood vessels, and macrophagic or neutrophilic infiltrate. The score assigned is 1–4 depending on the presentation. The final score has one extra point added for each dermal appendage (hair follicles, glands, or keratin) present. (**B**) Regenerative classification of skin: it has five regenerative stages (R0–R4), which are based on histological changes.

The assessment and application framework of the HEALS-A scale is specifically aimed at murine models of excisional wounds or other types of wounds where the healing process is by secondary intention, in which all layers of the skin have been removed. Complete removal of dermal tissue allows the assessment of all phases of wound healing and is also the ideal model to assess the recovery of skin structures at the wound site, reflecting tissue regeneration. The regeneration of skin structures is a major challenge for regenerative wound treatments; however, the HEALS-A scale will allow differentiation between the presence of only scar tissue at the wound site or the partial or complete recovery of skin structures and appendages (see Figure 3).

In HEALS-A, each characteristic is assigned a score from 1 to 4 depending on the degree of presence (Figure 3A). In addition, an extra point is assigned if the evaluated tissue presents skin appendages such as hair follicles, granules, or keratin. The presence of these structures shows the regeneration of complex structures that favor the restoration of skin functions. The total sum of the points for each of the characteristics reflects the degree of skin regeneration.

The classification consists of five stages, from R0 to R4, which outline the progression of wound healing (see Figure 3B). These stages, described below, capture key aspects of the wound healing process:

- R0: The basal stage (7 points) corresponds to a recently formed wound and/or skin without regeneration.
- R1: Incipient regeneration (8–10 points), which shows the early stages of skin repair.
- R2: Moderate regeneration (11–15 points) is characterized by noticeable tissue recovery.
- R3: Consolidated regeneration (16–20 points). This stage reflects advanced healing and tissue formation.
- R4: Complete regeneration (21–23 points). The skin appears fully healed or resembles perilesional skin (Figure 3B).

To illustrate the use of the HEALS-A scale, consider the following example: epithelium with development less than 25% (1 point) + slight angiogenesis (2 points) + slight neutrophilic inflammatory infiltrate (3 points) + moderate macrophagic inflammatory infiltrate (2 points) + scar tissue less than 25% (1 point), without the presence of skin appendages is equal to 9 points, which corresponds to stage R1 skin with incipient regeneration. In this case, the wound may be in an intermediate phase between the inflammatory and proliferative phases, based on the histological data observed in the example [58,59]. The degree of regeneration can be varied progressively over time. However, skin regeneration will depend on factors such as individual response, underlying diseases, and genetic variability, although it will largely depend on the response to treatment. Some treatments can achieve complete regeneration in short periods, while others may take longer. Histological evaluation using HEALS-A can identify different degrees of regeneration, but assessment times vary depending on each model. It is therefore important to remember that the healing process is dynamic and that histological assessment only reflects the state of the tissue at a specific time. The histological assessment can be achieved if other wound characteristics are considered, allowing for an evaluation and the establishment of appropriate timing for histological analysis. Clinical assessments are an integral part of this comprehensive evaluation and can be performed continuously to identify changes over time and in various wound characteristics, as will be described in the next section.

### 2.2. Clinical Evaluation Scale for Skin Regeneration (GRADES)

**Figure 4:**
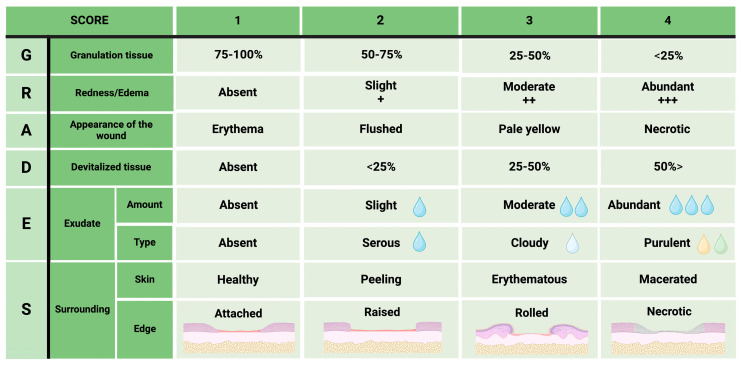
Clinical scale of skin regeneration (GRADES). The following clinical aspects are evaluated: wound appearance, amount of exudate, type of exudate, necrotic or detached tissue, granulation tissue, edema, perilesional skin, and wound edges. The score assigned is from 1 to 4 depending on the presentation. The total sum of the points reflects the clinical condition of the wound.

There are different ways of classifying wounds: they can be classified according to their type, evolution time, triggering mechanism, location, and contamination, among others [60,61]. However, all wounds present the four phases of the healing process described above. The differences between one wound and another are the time and the underlying factors preventing progress from one phase to another until wound closure [62]. Building on this understanding, we developed a clinical scale, referred to as GRADES: an acronym of granulation tissue, redness/edema, appearance of wound, devitalized tissue (necrotic/sloughed), exudate type/amount, and surrounding skin (Figure 4). The GRADES scale was adapted from clinical assessments in humans [6,7,8,9,63] and evaluates clinical data that reflect the phases of the healing process such as exudate, which is a clinical characteristic of the hemostatic–inflammatory phase [64], edema of the inflammatory phase [65], and granulation tissue of the proliferative–remodeling phase [66]. However, some clinical data may reflect complications such as the appearance of the wound and necrotic/sloughed tissue from tissue damage [23], type of exudate, appearance of the wound, and edema from infections [67]. Finally, the state of the perilesional skin [68] and the type of wound edge help decision making during wound monitoring [69]. The score of this scale ranges from 1 to 4 points, depending on the clinical presentation (Figure 4).

**Figure 5:**
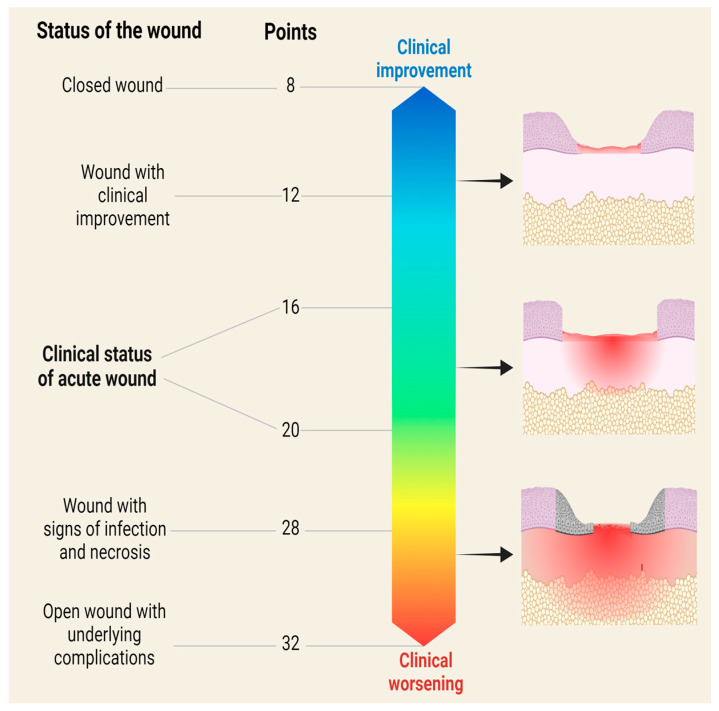
Clinical classification of the skin’s regenerative process. The baseline clinical status of a newly appearing wound is 16–20 points. A decrease in the baseline score indicates clinical improvement and an increase in the baseline score indicates clinical worsening.

The total score, obtained by adding the points for each clinical aspect, indicates whether the wound is clinically improving or worsening (Figure 5). To illustrate the use of the GRADES scale, consider this example: granulation tissue: <25% (4 points) + redness/edema: moderate (3 points) + wound appearance: erythema (1 point) + devitalized tissue: <25% (2 points) + exudate: moderate (3 points) + exudate: cloudy (3 points) + surrounding skin: erythematous (3 points) + edge: raised (2 points). The sum of the points for each characteristic is equal to 21 points. If the wound initially had an average score (16–20 points), the current score indicates clinical deterioration, requiring the presence of an infectious process to be ruled out and appropriate decisions to be made accordingly. Preclinical models of whole-thickness skin wounds measured with this scale usually present a baseline score ranging from 16 to 20 points. However, a decrease in the score relative to the baseline suggests clinical improvement (for example, a score as low as 8 points might be a healed or almost-healed wound), whereas an increase in the score suggests clinical worsening, which could be associated with infections, adverse reactions to treatment, rejection responses, underlying problems specific to the animal, or procedural errors during wound generation or follow-up (Figure 5).

## 3. Discussion

The preclinical evaluation of a wound model is based on three key pillars: wound closure with respect to the time of evolution, the histological evaluation of the skin regeneration at the wound site, and the clinical response of the wound to treatment. However, as far as we know most investigations only evaluate one of the three pillars described [20,70,71,72]. Previous evaluations are also based on histological parameters such as granulation tissue, quantity, maturation and orientation of collagen fibers or extracellular matrix proteins, inflammatory infiltrate, and new blood vessels [11,12,13,15]. Other assessments are based on criteria like re-epithelialization, epidermal thickness, granulation tissue, and dermal remodeling, with the analysis of these histological aspects being performed using specialized software [14]. However, to date there is not a consensus regarding the characteristics to evaluate.

**Figure 6:**
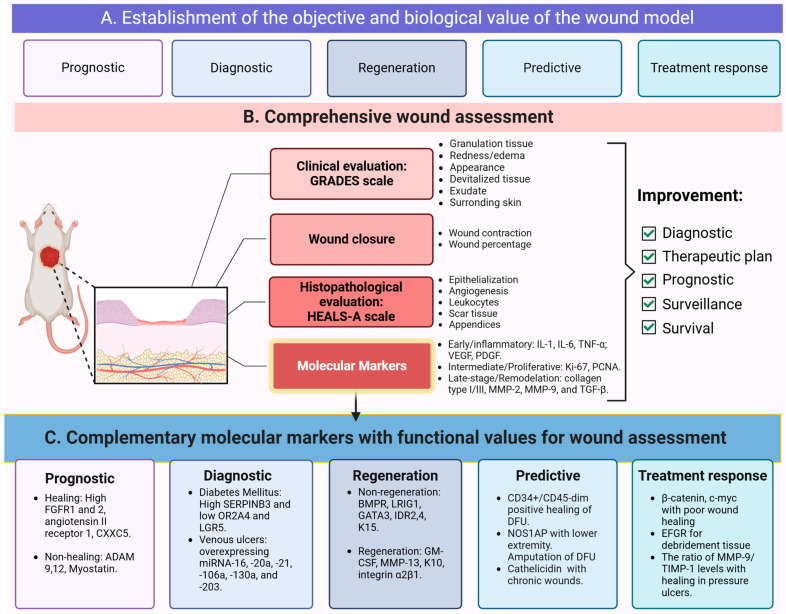
Biological value of a wound model and its comprehensive assessment. Wounds in preclinical models should be evaluated from multiple perspectives. The first step involves identifying the biological value of the selected wound model (**A**). Subsequently, a comprehensive evaluation should be conducted macroscopically through wound closure and clinical response (GRADES), and microscopically through histological analysis with tools such as HEALS-A (**B**). The data obtained from these evaluations can guide decisions in wound management, such as adjustments to therapeutic formulations, improvements in animal care, or modifications to experimental protocols. Various types of molecular markers are integral to wound assessment (**C**). Researchers must clearly define the purpose of each molecule to effectively integrate molecular marker results with other findings (A-C). IL-1 and IL-6: Interleukin-1 and -6, TNF-α: Tumor Necrosis Factor-alpha, VEGF: Vascular Endothelial Growth Factor, PDGF: Platelet-Derived Growth Factor, FGFR1 and 2: Fibroblast Growth Factor Receptor 1 and 2, CXXC5: CXXC-Type Zinc Finger Protein 5, ADAM9 and 12: A Disintegrin and Metalloprotease 9 and 12, SERPINB3: Serpin Peptidase Inhibitor, Clade B, Member 3, OR2A4: Olfactory Receptor Family 2 Subfamily A Member 4, LGR5: Leucine-Rich Repeat-Containing G-Protein Coupled Receptor 5, microRNAs: miRNAs, BMPR: Bone Morphogenetic Protein Receptor, LRIG1: Leucine-Rich Repeats and Immunoglobulin-Like Domains Protein 1, GATA3: GATA Binding Protein 3, ID2 and ID4: Inhibitors of DNA-Binding Proteins 2 and 4, KRT10 and 15: Keratin 10 and 15, GM-CSF2: Granulocyte-Macrophage Colony-Stimulating Factor 2, MMP-13: Matrix Metalloproteinase-13, ITGA2B1: Integrin α2β1, CD34/CD45: Cluster of differentiation 34/45, NOS1AP: nitric oxide synthase 1 adaptor protein, DFU: diabetic foot ulcer, c-myc: cellular myelocytomatosis homologous viral oncogene protein, EGFR: Epidermal growth factor, MMP-13: Matrix Metalloproteinase-13, TIMP-1: TIMP Metallo-peptidase Inhibitor 1.

In this paper, we proposed two novel evaluation scales for assessing skin regeneration in murine wound healing models: the histological HEALS-A scale and the clinical GRADES scale. These scales may complement and favor a comprehensive evaluation of the wounds in preclinical models. This comprehensive assessment ideally should be based on clinical characteristics, wound closure data, as well as histological evaluation, which facilitate the appropriate selection of molecular markers for evaluation and/or follow-up. This approach enhances the understanding of wound treatment and provides a more-accurate reflection of the observed treatment response. Figure 6 presents an integral evaluation of wounds, including the clinical and histological scales proposed in this paper, along with the timing of wound closure through software, and validated markers identified in the literature, classified according to their biological relevance.

The GRADES score is particularly advantageous and novel for clinical wound assessment as it allows for real-time and non-invasive assessment of wound characteristics, focusing on clinically relevant parameters that can be properly measured in animals, such as the presence and extent of granulation tissue, signs of redness or edema indicative of inflammation, the overall appearance of the wound denoting vascularity, cleanliness, and progression, and the identification of devitalized or necrotic tissue that may impede healing and promote complications. This score can provide a standardized method for monitoring wound status at different time points, allowing researchers to track dynamic changes without requiring invasive procedures and also have the power to make decisions about wound management. The GRADES score assesses clinical aspects very similar to those assessed in humans. This score can be applied to other wound models, making it an essential tool for clinical wound assessment in animals. The GRADES clinical scale proposed is an adaptation of different clinical scales validated for humans [6,7,8]. However, our adaptation includes a staging of the clinical characteristics. None of the reference scales stage the clinical aspects evaluated. This distinction highlights GRADES as a more-effective alternative to reference scales, making it the ideal tool for wound assessment across various animal models. Moreover, histological evaluation is widely recognized as an effective method for demonstrating changes in skin structures, enabling the identification of the healing phase and/or potential dermal regeneration [73,74]. However, the assessment of the histopathological status of wounds can be complemented by using this new tool in conjunction with existing histological scales, as each describes different wound characteristics. The HEALS-A scale has a regenerative approach that focuses on the recovery of dermal structures and thus skin functions. Tissue assessment using the HEALS-A scale provides valuable insights into the degree of dermal structure recovery at the wound site. In cases where complete skin regeneration does not occur, detailed characterization of the collagen structures—such as their orientation, patterns, quantity, and changes—offers a comprehensive depiction of the scar tissue. Both the HEALS-A and GRADES scales can be used without the need for specialized software; however, the implementation of HEALS-A should be carried out by a veterinary pathologist.

In facilities equipped with the necessary infrastructure and high-resolution imaging devices, as well as trained personnel proficient in software such as ImageJ vesion 1.52i, a more-advanced analysis of tissue images can be conducted. This includes evaluating the thickness and volume of the epidermis and dermis at the wound site. Such technological integration enables a comprehensive histological evaluation using multiple approaches and diverse assessment methods, as seen in Figure 6B. Similarly, the inclusion of molecular markers in wound evaluation should be carried out systematically [75,76]. In this context, selecting an appropriate molecular marker requires the researcher to clearly define its purpose to effectively integrate the molecular marker results with other clinical outcomes (Figure 6A–C). This systematic approach provides a robust and multidimensional framework for wound assessment, combining the practical advantages of clinical observation with the detailed insights of histological analysis. Moreover, the integration of wound closure data with clinical, histological, and molecular findings enhances the overall assessment, ultimately supporting the development and validation of new treatments in preclinical studies.

Finally, it is important to mention that the application of both scales may be extended to other types of injuries such as chronic or infected wounds. These types of wounds have complex pathophysiological characteristics; however, the regeneration of the dermal structures and their recovery will depend on the correct treatment of underlying diseases, the individual’s own response, the treatment applied to the wound, and the wound microbiome, among others. These two types of wounds can be evaluated with the proposed scales, but the scores of both will be different from those of an excisional acute wound. However, based on an initial score of these wounds, subsequent evaluations can be carried out to reflect whether the wounds tend to display clinical improvement and the regeneration of structures after their treatment or not. However, in order to confirm this experimental results would be needed to support their applicability to this kind of wounds.

## 4. Conclusions

The histological (HEALS-A) and clinical (GRADES) scales proposed in this study provide tools for evaluating skin regeneration in preclinical murine wound healing models. By integrating macroscopic clinical observations with microscopic histological insights, these scales provide a robust and systematic methodology to assess wound healing progression. The GRADES scale allows for real-time monitoring of wound outcomes, while the HEALS-A scale captures critical cellular and extracellular events essential for tissue repair. Together, they address some limitations of existing methods by offering a complementary and multidimensional perspective. These tools will enable us to effectively identify the regeneration of anatomical structures in the skin, rather than merely assessing the type of scarring present in the injured skin. Staging the regenerative state of the skin will improve the evaluation of therapeutic outcomes from new treatments and foster the development of more-effective therapies for wounds or injuries in other tissues.

## Data Availability

No new data were created or analyzed in this study. Data sharing is not applicable to this article.

## References

[1] Afonso A.C., Oliveira D., Saavedra M.J., Borges A., Simoes M. (2021). Biofilms in Diabetic Foot Ulcers: Impact, Risk Factors and Control Strategies. Int. J. Mol. Sci..

[2] Kujath P., Michelsen A. (2008). Wounds—From physiology to wound dressing. Dtsch. Arztebl. Int..

[3] Maheshwari G. (2024). Chronic wounds: A rising public health concern. J. Wounds APAC.

[4] Kolimi P., Narala S., Nyavanandi D., Youssef A.A.A., Dudhipala N. (2022). Innovative Treatment Strategies to Accelerate Wound Healing: Trajectory and Recent Advancements. Cells.

[5] Park J.C., Lee D.H., Suh H. (1999). Preclinical evaluation of prototype products. Yonsei Med. J..

[6] Lumbers M. (2019). TIMERS: Undertaking wound assessment in the community. Br. J. Community Nurs..

[7] Valenzuela A., Prieto E. (2020). El acrónimo “DOMINATE” como instrumento necesario en el tratamiento de un paciente con una úlcera venosa. Rev. Enfermería Vasc..

[8] Gale S.S., Lurie F., Treadwell T., Vazquez J., Carman T., Partsch H., Alvarez O., Langemo D., Posthauer M.E., Cheney M. (2014). DOMINATE Wounds. Wounds Compend. Clin. Res. Pract..

[9] Gil S.B. (2020). Implementing the Triangle of Wound Assessment framework to transform the care pathway for diabetic foot ulcers. J. Wound Care.

[10] Greco A., Mastronicola D., Pacini F., Giacomelli L., Papa S., Fiorentini C., David V., Rowan S., Mennini N., Magnoni C. (2023). Researching the level of agreement among experts on terms used to describe wounds: An international study. Int. Wound J..

[11] Sami D.G., Abdellatif A. (2020). Histological and clinical evaluation of wound healing in pressure ulcers: A novel animal model. J. Wound Care.

[12] Santos T.S., Santos I., Pereira-Filho R.N., Gomes S.V.F., Lima-Verde I.B., Marques M.N., Cardoso J.C., Severino P., Souto E.B., Albuquerque-Junior R.L.C. (2021). Histological Evidence of Wound Healing Improvement in Rats Treated with Oral Administration of Hydroalcoholic Extract of Vitis labrusca. Curr. Issues Mol. Biol..

[13] Deyhimi P., Khademi H., Birang R., Akhoondzadeh M. (2016). Histological Evaluation of Wound Healing Process after Photodynamic Therapy of Rat Oral Mucosal Ulcer. J. Dent..

[14] van de Vyver M., Boodhoo K., Frazier T., Hamel K., Kopcewicz M., Levi B., Maartens M., Machcinska S., Nunez J., Pagani C. (2021). Histology Scoring System for Murine Cutaneous Wounds. Stem Cells Dev..

[15] Sultana J., Molla M.R., Kamal M., Shahidullah M., Begum F., Bashar M.A. (2009). Histological differences in wound healing in Maxillofacial region in patients with or without risk factors. Bangladesh J. Pathol..

[16] Peyron P.A., Colomb S., Becas D., Adriansen A., Gauchotte G., Tiers L., Marin G., Lehmann S., Baccino E., Delaby C. (2021). Cytokines as new biomarkers of skin wound vitality. Int. J. Leg. Med..

[17] Duan M., Zhang Y., Zhang H., Meng Y., Qian M., Zhang G. (2020). Epidermal stem cell-derived exosomes promote skin regeneration by downregulating transforming growth factor-β1 in wound healing. Stem Cell Res. Ther..

[18] Masson-Meyers D.S., Andrade T.A.M., Caetano G.F., Guimaraes F.R., Leite M.N., Leite S.N., Frade M.A.C. (2020). Experimental models and methods for cutaneous wound healing assessment. Int. J. Exp. Pathol..

[19] Shao K., Han B., Gao J., Jiang Z., Liu W., Liu W., Liang Y. (2016). Fabrication and feasibility study of an absorbable diacetyl chitin surgical suture for wound healing. J. Biomed. Mater. Res. Part B Appl. Biomater..

[20] Yampolsky M., Bachelet I., Fuchs Y. (2024). Reproducible strategy for excisional skin-wound-healing studies in mice. Nat. Protoc..

[21] Larouche J., Sheoran S., Maruyama K., Martino M.M. (2018). Immune Regulation of Skin Wound Healing: Mechanisms and Novel Therapeutic Targets. Adv. Wound Care.

[22] Szpaderska A.M., Egozi E.I., Gamelli R.L., DiPietro L.A. (2003). The effect of thrombocytopenia on dermal wound healing. J. Investig. Dermatol..

[23] Weiss S.J. (1989). Tissue destruction by neutrophils. N. Engl. J. Med..

[24] Brinkmann V., Reichard U., Goosmann C., Fauler B., Uhlemann Y., Weiss D.S., Weinrauch Y., Zychlinsky A. (2004). Neutrophil extracellular traps kill bacteria. Science.

[25] Kolaczkowska E., Kubes P. (2013). Neutrophil recruitment and function in health and inflammation. Nat. Rev. Immunol..

[26] Devalaraja R.M., Nanney L.B., Du J., Qian Q., Yu Y., Devalaraja M.N., Richmond A. (2000). Delayed wound healing in CXCR2 knockout mice. J. Investig. Dermatol..

[27] Dovi J.V., He L.K., DiPietro L.A. (2003). Accelerated wound closure in neutrophil-depleted mice. J. Leukoc. Biol..

[28] Agren M.S., Jorgensen L.N., Andersen M., Viljanto J., Gottrup F. (1998). Matrix metalloproteinase 9 level predicts optimal collagen deposition during early wound repair in humans. Br. J. Surg..

[29] Brown B.N., Londono R., Tottey S., Zhang L., Kukla K.A., Wolf M.T., Daly K.A., Reing J.E., Badylak S.F. (2012). Macrophage phenotype as a predictor of constructive remodeling following the implantation of biologically derived surgical mesh materials. Acta Biomater..

[30] Willenborg S., Eming S.A. (2014). Macrophages—Sensors and effectors coordinating skin damage and repair. J. Ger. Soc. Dermatol..

[31] Wallace H.A., Basehore B.M., Zito P.M. (2023). Wound Healing Phases. [Updated 2023 Jun 12]. StatPearls [Internet].

[32] Schultz G.S., Chin G.A., Moldawer L., Diegelmann R.F., Fitridge R., Thompson M. (2011). Principles of wound healing. Mechanisms of Vascular Disease: A Reference Book for Vascular Specialists.

[33] Eming S.A., Krieg T., Davidson J.M. (2007). Inflammation in wound repair: Molecular and cellular mechanisms. J. Investig. Dermatol..

[34] Eming S.A., Martin P., Tomic-Canic M. (2014). Wound repair and regeneration: Mechanisms, signaling, and translation. Sci. Transl. Med..

[35] Grellner W. (2002). Time-dependent immunohistochemical detection of proinflammatory cytokines (IL-1β, IL-6, TNF-alpha) in human skin wounds. Forensic Sci. Int..

[36] Mast B.A., Schultz G.S. (1996). Interactions of cytokines, growth factors, and proteases in acute and chronic wounds. Wound Repair Regen. Off. Publ. Wound Heal. Soc. Eur. Tissue Repair Soc..

[37] Olczyk P., Mencner L., Komosinska-Vassev K. (2014). The role of the extracellular matrix components in cutaneous wound healing. BioMed Res. Int..

[38] Lindley L.E., Stojadinovic O., Pastar I., Tomic-Canic M. (2016). Biology and Biomarkers for Wound Healing. Plast. Reconstr. Surg..

[39] Gurtner G.C., Werner S., Barrandon Y., Longaker M.T. (2008). Wound repair and regeneration. Nature.

[40] Pastar I., Stojadinovic O., Yin N.C., Ramirez H., Nusbaum A.G., Sawaya A., Patel S.B., Khalid L., Isseroff R.R., Tomic-Canic M. (2014). Epithelialization in Wound Healing: A Comprehensive Review. Adv. Wound Care.

[41] Ramirez H.A., Liang L., Pastar I., Rosa A.M., Stojadinovic O., Zwick T.G., Kirsner R.S., Maione A.G., Garlick J.A., Tomic-Canic M. (2015). Comparative Genomic, MicroRNA, and Tissue Analyses Reveal Subtle Differences between Non-Diabetic and Diabetic Foot Skin. PLoS ONE.

[42] Pastar I., Khan A.A., Stojadinovic O., Lebrun E.A., Medina M.C., Brem H., Kirsner R.S., Jimenez J.J., Leslie C., Tomic-Canic M. (2012). Induction of specific microRNAs inhibits cutaneous wound healing. J. Biol. Chem..

[43] Lee S.H., Kim M.Y., Kim H.Y., Lee Y.M., Kim H., Nam K.A., Roh M.R., Min D.S., Chung K.Y., Choi K.Y. (2015). The Dishevelled-binding protein CXXC5 negatively regulates cutaneous wound healing. J. Exp. Med..

[44] Meyer M., Muller A.K., Yang J., Moik D., Ponzio G., Ornitz D.M., Grose R., Werner S. (2012). FGF receptors 1 and 2 are key regulators of keratinocyte migration in vitro and in wounded skin. J. Cell Sci..

[45] Takeda H., Katagata Y., Hozumi Y., Kondo S. (2004). Effects of angiotensin II receptor signaling during skin wound healing. Am. J. Pathol..

[46] Zhang C., Tan C.K., McFarlane C., Sharma M., Tan N.S., Kambadur R. (2012). Myostatin-null mice exhibit delayed skin wound healing through the blockade of transforming growth factor-β signaling by decorin. Am. J. Physiol. Cell Physiol..

[47] Harsha A., Stojadinovic O., Brem H., Sehara-Fujisawa A., Wewer U., Loomis C.A., Blobel C.P., Tomic-Canic M. (2008). ADAM12: A potential target for the treatment of chronic wounds. J. Mol. Med..

[48] Mauch C., Zamek J., Abety A.N., Grimberg G., Fox J.W., Zigrino P. (2010). Accelerated wound repair in ADAM-9 knockout animals. J. Investig. Dermatol..

[49] Stojadinovic O., Pastar I., Nusbaum A.G., Vukelic S., Krzyzanowska A., Tomic-Canic M. (2014). Deregulation of epidermal stem cell niche contributes to pathogenesis of nonhealing venous ulcers. Wound Repair Regen. Off. Publ. Wound Heal. Soc. Eur. Tissue Repair Soc..

[50] Xie J.L., Li T.Z., Qi S.H., Bian H.N., Cheng J.D., Xu Y.B., Liang H.Z. (2003). [A preliminary study on the identification and distribution of epidermal stem cells in different degrees of burn wounds in scalded rats]. Zhonghua Shao Shang Za Zhi Zhonghua Shaoshang Zazhi = Chin. J. Burn..

[51] Stacey M.C. (2020). Biomarker directed chronic wound therapy—A new treatment paradigm. J. Tissue Viability.

[52] Yang R., Wang J., Chen X., Shi Y., Xie J. (2020). Epidermal Stem Cells in Wound Healing and Regeneration. Stem Cells Int..

[53] Thom S.R., Hampton M., Troiano M.A., Mirza Z., Malay D.S., Shannon S., Jennato N.B., Donohue C.M., Hoffstad O., Woltereck D. (2016). Measurements of CD34+/CD45-dim Stem Cells Predict Healing of Diabetic Neuropathic Wounds. Diabetes.

[54] Margolis D.J., Hampton M., Hoffstad O., Mala D.S., Mirza Z., Woltereck D., Shannon S., Troiano M.A., Mitra N., Yang M. (2017). NOS1AP genetic variation is associated with impaired healing of diabetic foot ulcers and diminished response to healing of circulating stem/progenitor cells. Wound Repair Regen. Off. Publ. Wound Heal. Soc. Eur. Tissue Repair Soc..

[55] Stojadinovic O., Brem H., Vouthounis C., Lee B., Fallon J., Stallcup M., Merchant A., Galiano R.D., Tomic-Canic M. (2005). Molecular pathogenesis of chronic wounds: The role of β-catenin and c-myc in the inhibition of epithelialization and wound healing. Am. J. Pathol..

[56] Heilborn J.D., Nilsson M.F., Kratz G., Weber G., Sorensen O., Borregaard N., Stahle-Backdahl M. (2003). The cathelicidin anti-microbial peptide LL-37 is involved in re-epithelialization of human skin wounds and is lacking in chronic ulcer epithelium. J. Investig. Dermatol..

[57] Ladwig G.P., Robson M.C., Liu R., Kuhn M.A., Muir D.F., Schultz G.S. (2002). Ratios of activated matrix metalloproteinase-9 to tissue inhibitor of matrix metalloproteinase-1 in wound fluids are inversely correlated with healing of pressure ulcers. Wound Repair Regen. Off. Publ. Wound Heal. Soc. Eur. Tissue Repair Soc..

[58] Koh T.J., DiPietro L.A. (2011). Inflammation and wound healing: The role of the macrophage. Expert Rev. Mol. Med..

[59] DiPietro L.A., Polverini P.J. (1993). Role of the macrophage in the positive and negative regulation of wound neovascularization. Behring Inst. Mitteilungen.

[60] Iruela Sanchez M., García-Sierra G., Llado Blanch M., Naveros Almenara F., Seda G., Toran-Monserrat P. (2023). Typology of wounds treated in primary care: Multicenter cross-sectional study. Semergen.

[61] Herman T.F., Popowicz P., Bordoni B. (2024). Wound Classification. StatPearls.

[62] Vallejo L. (2020). Seven common errors in the diagnosis, management and treatment of chronic wounds. J. Wound Care.

[63] Leal C.A.I., Morgado P. (2000). Manejo y tratamiento de las heridas (Valoración y clasificación). Guía Clin. N°1 Del MINSAL.

[64] World Union of Wound Healing Societies (2007). Principles of Best Practice: Wound Exudate and the Role of Dressings. A Consensus Document.

[65] Villeco J.P. (2012). Edema: A silent but important factor. J. Hand Ther. Off. J. Am. Soc. Hand Ther..

[66] Alhajj M.G.A. (2024). Physiology, Granulation Tissue. StatPearls.

[67] Healy B., Freedman A. (2006). Infections. Bmj.

[68] Rippon M.G., Rogers A.A., Ousey K., Atkin L., Williams K. (2022). The importance of periwound skin in wound healing: An overview of the evidence. J. Wound Care.

[69] Dodds S., Hayes S. (2004). The wound edge, epithelialization and monitoring wound healing. A journey through TIME. Wound bed preparation in practice. Br. J. Nurs. Suppl..

[70] de Vasconcelos Catao M.H., Nonaka C.F., de Albuquerque R.L., Bento P.M., de Oliveira Costa R. (2015). Effects of red laser, infrared, photodynamic therapy, and green LED on the healing process of third-degree burns: Clinical and histological study in rats. Lasers Med. Sci..

[71] Hoisang S., Kampa N., Seesupa S., Jitpean S. (2021). Assessment of wound area reduction on chronic wounds in dogs with photobiomodulation therapy: A randomized controlled clinical trial. Vet. World.

[72] Lucroy M.D., Edwards B.F., Madewell B.R. (1999). Low-intensity laser light-induced closure of a chronic wound in a dog. Vet. Surg..

[73] Balderas-Cordero D., Canales-Alvarez O., Sánchez-Sánchez R., Cabrera-Wrooman A., Canales-Martinez M.M., Rodriguez-Monroy M.A. (2023). Anti-Inflammatory and Histological Analysis of Skin Wound Healing through Topical Application of Mexican Propolis. Int. J. Mol. Sci..

[74] Tottoli E.M., Dorati R., Genta I., Chiesa E., Pisani S., Conti B. (2020). Skin Wound Healing Process and New Emerging Technologies for Skin Wound Care and Regeneration. Pharmaceutics.

[75] Califf R.M. (2018). Biomarker definitions and their applications. Exp. Biol. Med..

[76] Yang J., Schiffer C.A. (2012). Genetic biomarkers in acute myeloid leukemia: Will the promise of improving treatment outcomes be realized?. Expert Rev. Hematol..

